# Uptake and Release of Species from Carbohydrate Containing Organogels and Hydrogels

**DOI:** 10.3390/gels5040043

**Published:** 2019-09-30

**Authors:** Abhishek Pan, Saswati G. Roy, Ujjal Haldar, Rita D. Mahapatra, Garry R. Harper, Wan Li Low, Priyadarsi De, John G. Hardy

**Affiliations:** 1 Polymer Research Centre and Centre for Advanced Functional Materials, Department of Chemical Sciences, Indian Institute of Science Education and Research Kolkata, Mohanpur Nadia 741246, West Bengal, India; abhishekpan99@gmail.com (A.P.); saswati.ghoshroy9@gmail.com (S.G.R.); haldar.ujjal10@gmail.com (U.H.); rita_das@chem.iitkgp.ernet.in (R.D.M.); 2 Department of Chemistry, Lancaster University, Lancaster, Lancashire LA1 4YB, UK; garryharper3@gmail.com; 3 School of Pharmacy, University of Wolverhampton, Wolverhampton WV1 1LY, UK; 4 Materials Science Institute, Lancaster University, Lancaster, Lancashire, LA1 4YB, UK

**Keywords:** RAFT, organogel, hydrogel, crosslinking, swelling, uptake, release

## Abstract

Hydrogels are used for a variety of technical and medical applications capitalizing on their three-dimensional (3D) cross-linked polymeric structures and ability to act as a reservoir for encapsulated species (potentially encapsulating or releasing them in response to environmental stimuli). In this study, carbohydrate-based organogels were synthesized by reversible addition-fragmentation chain transfer (RAFT) polymerization of a *β*-D-glucose pentaacetate containing methacrylate monomer (Ac-glu-HEMA) in the presence of a di-vinyl cross-linker; these organogels could be converted to hydrogels by treatment with sodium methoxide (NaOMe). These materials were studied using solid state ^13^C cross-polarization/magic-angle spinning (CP/MAS) NMR, Fourier transform infrared (FTIR) spectroscopy, and field emission scanning electron microscopy (FE-SEM). The swelling of the gels in both organic solvents and water were studied, as was their ability to absorb model bioactive molecules (the cationic dyes methylene blue (MB) and rhodamine B (RhB)) and absorb/release silver nitrate, demonstrating such gels have potential for environmental and biomedical applications.

## 1. Introduction

The fundamental science and engineering that underpins the development of gels with customizable properties and structures enables the production of materials with a variety of task-specific applications, potentially delivering beneficial economic, environmental, health and societal impacts [1,2,3,4,5,6,7].

Materials science underpins products worth billions of dollars and directly/indirectly supports millions of jobs worldwide. Gels are a class of materials with properties suitable for a variety of technical and medical applications [1,2,3,4,5,6,7] that capitalize on their three-dimensional (3D) cross-linked polymeric structures and ability to act as a reservoir for encapsulated species. Gels can be broadly divided into two groups, organogels and hydrogels, depending on the liquid immobilized in their 3D structures wherein the polymer chains are crosslinked by covalent chemical bonds or non-covalent physical interactions [1,2,3,4,5,6,7]. Gels can encapsulate and/or release species, potentially in response to externally applied stimuli [8], which offers opportunities for application as drug/fragrance delivery systems [9], hand sanitizers [10], and recovery/separation of crude oil [11,12,13]. Gels are the subject of a multitude of review articles, and a comprehensive review of the chemistry, engineering and physics of such materials is outside the scope of this communication, and the interested reader is directed towards a selection of insightful reviews [14,15,16,17,18].

Polysaccharides of natural and synthetic origins are a common component of gels used for biomedical applications because they tend to be cheap and their properties are easily tuned; moreover, they tend to be relatively non-immunogenic which is important for biomedical applications [19,20,21,22,23].

Herein we report the preparation of carbohydrate-based gels via the reversible-addition fragmentation chain transfer (RAFT) polymerization [24,25] of *β*-D-glucose pentaacetate containing methacrylate (Ac-glu-HEMA) monomer in the presence of di(ethylene glycol) dimethacrylate (DEGDMA) as a divinyl cross-linker (yielding organogels), optionally followed by a simple one step deprotection of the acetyl groups by base hydrolysis (yielding hydrogels). The uptake and release of species from the gels was demonstrated, showing such gels have potential environmental and biomedical applications.

## 2. Results and Discussion

### 2.1. Gel Chemistry

**Scheme 1:**
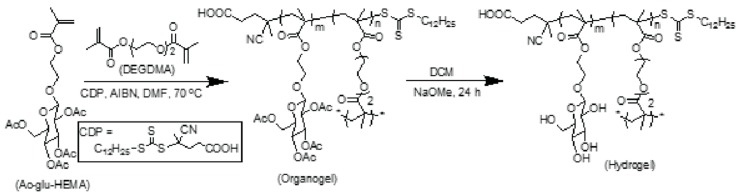
Preparation of carbohydrate-based organogels via reversible-addition fragmentation chain transfer (RAFT) polymerization, and the optional conversion to hydrogels by deprotection of the acetyl groups.

We have previously reported that 4-cyano-4-(dodecylsulfanylthiocarbonyl)sulfanylpentanoic acid (CDP) acts as an efficient chain transfer agent (CTA) for the RAFT polymerization of methacrylated carbohydrates at 70 °C in DMF [26]. Herein we investigated the polymerization of mixtures of 2-hydroxyethyl methacrylate (HEMA) functionalized and acetyl (Ac) protected glucose derivative (Ac-glu-HEMA) in the presence of the di(ethylene glycol) dimethacrylate (DEGDMA) cross-linker at different Ac-glu-HEMA/DEGDMA ratios, using 2,2’-azobisisobutyronitrile (AIBN) and CDP in DMF at 70 °C, that could be converted to hydrogels via deprotection of the acetyl groups (Scheme 1), yielding organogels and hydrogels with high swelling ratios and uniform pore structures.

**Figure 1:**
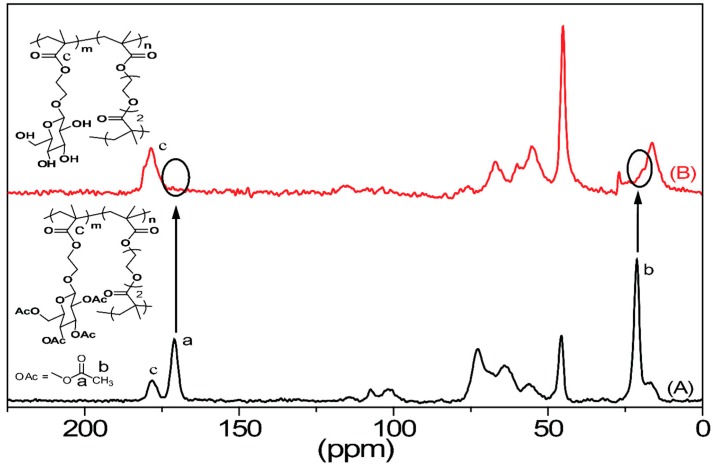
Representative solid state ^13^C CP/MAS NMR spectra of C200 gel before (**A**) and after (**B**) acetyl (-OAc) group deprotection.

**Figure 2:**
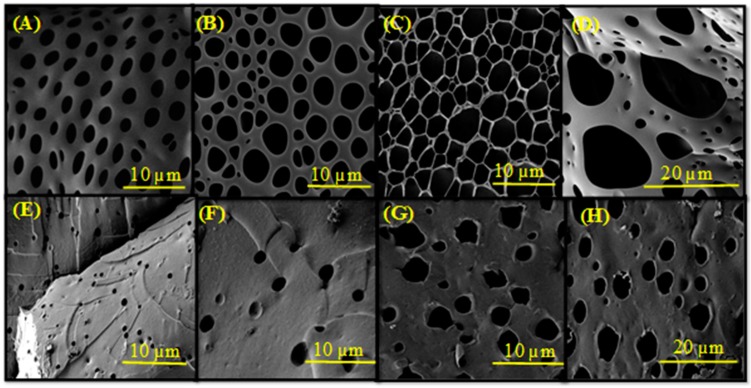
FE-SEM micrographs of gels. (**A**) C50; (**B**) C100; (**C**) C200; (**D**) C300; (**E**) DC50; (**F**) DC100; (**G**) DC200; and (**H**) DC300.

The organogels were prepared by changing the [Ac-glu-HEMA]/[DEGDMA] ratios from 50/2 to 300/2, keeping CDP and AIBN concentrations constant. Preliminary experiments indicated that the amount of time necessary to achieve gelation was observed to increase with increasing Ac-glu-HEMA/DEGDMA ratios from 50/2 to 300/2, as summarized in Table 1. Since RAFT gelation proceeds through fast initiation followed by slow propagation [27], we carried out all subsequent studies using gels after reaction for 24 h to ensure the maximum monomer conversion (estimated gravimetrically by comparing the weight of the dry gels with respect to the monomer feed, as summarized in Table 1). The compositions of the feed mixtures of the organogels are listed in Table 1. The letter ‘C’ stands for carbohydrate; for the hydrogels generated by acetyl group deprotection of the organogels, the letter ‘D’ was introduced before the letter C (i.e., C50 becomes DC50 after deprotection of the acetyls). The organogels had a faint yellow color indicative of the retention of the trithiocarbonate functionality in the 3D matrix, and their transparency decreases from C50 to C300. Deprotection of the acetyl groups from the organogels was achieved by swelling them in DCM overnight followed by treatment with NaOMe at room temperature. The hydrogels (DC50–DC300) were translucent when hydrated in water. The dried organogels and hydrogels were characterized by solid state ^13^C CP/MAS NMR (Figure 1) and FTIR spectroscopy (Figure 2).

**Figure A1:**
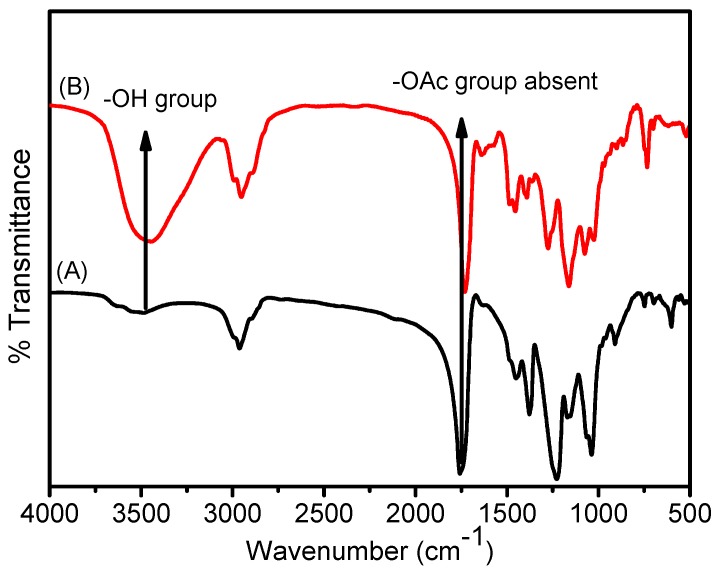
FTIR spectra. (**A**) Dried C200 organogel. (**B**) Dried DC200 hydrogel.

Solid state ^13^C CP/MAS NMR of the C200 organogel (Figure 1A) displays characteristic resonance signals from different carbon atoms in the sugar and polymer backbone (although carbon peaks from DEGDMA and CDP moiety were not easily visible, due to their low abundance). Importantly, signals from the vinyl group of the Ac-glu-HEMA monomer were absent in Figure 1A, confirming the complete removal of unreacted Ac-glu-HEMA and DEGDMA from the gel after purification. Conversion of the C200 organogel (Figure 1A) to the DC200 hydrogel (Figure 1B) was demonstrated by the loss of the carbon signals at 170.2 and 21.1 ppm of the acetyl groups. FTIR data supported the conclusions drawn from the NMR data. The FTIR spectrum of the C200 organogel (Figure A1) showed strong vibrational stretching frequencies centered at 1166 and 1650–1830 cm^−1^ due to the C−O and carbonyl (C=O) groups, respectively. The broad absorption in the 2960–3000 cm^−1^ region was assigned to the aliphatic (Sp3) C–H stretching vibration modes. Peaks at 2921, 931, 1086 and 1030 cm^−1^ were due to the C–H (bending), C–O–C (asymmetric) and C–O–C (symmetric) stretching vibrations, respectively. The FTIR spectrum of the DC200 hydrogel (Figure A1) has a sharp broad band generated at 3516 cm^−1^ for the –OH stretching frequency, which also indicated successful removal of acetyl groups.

### 2.2. Macroscopic Properties of the Gels

**Figure 3:**
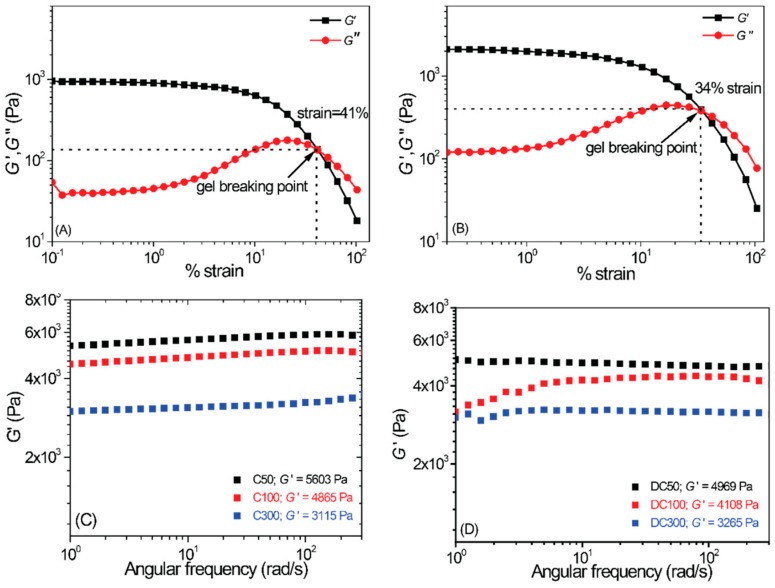
Storage modulus (G′) and loss modulus (G″) on strain sweep with the (**A**) C200 organogel and (**B**) DC200 hydrogel. Storage modulus G´ versus angular frequency (strain: 2.0%) of various (**C**) organogels and (**D**) hydrogels with different Ac-glu-HEMA/DEGDMA ratios.

The organogels and hydrogels were characterized by FE-SEM (Figure 2) and rheological measurements (Figure 3) that offer information about cross-linking density, structural homo-/heterogeneity and mechanics of the gel matrices. FE-SEM (Figure 2) indicated that the pore diameter of the organogels gradually increased with increasing the Ac-glu-HEMA/DEGDMA ratio from 50/2 to 300/2. Interestingly, the shapes of the pores changes from spherical round shaped (C50), to hexagonal honey comb (C200), to heterogeneous porous structures (C300), which is mainly regulated by the extent of cross-linking density. Decreasing cross-linking density increased the average pore diameters from 5.4 ± 1 µm (C50) to 8.5 ± 2 µm (C100) to 12.0 ± 1 µm (C200) to 20 ± 2 µm (C300) (Figure 2A–D). Similarly, the pore diameters of hydrogels also changed from 4.4 ± 1 µm (DC50) to 6.2 ± 2 µm (DC100) to 8.3 ± 2 µm (DC200) and 10.1 ± 2 µm (DC300) (Figure 2E–H). These results indicate that the internal porous structure of both organogels and hydrogels changes accordingly based on the density of cross-links.

Rheological studies (stress sweep and frequency sweep measurements) were conducted as a function of shear strain and frequency, respectively, for the organogels and hydrogels (Figure 3). For the organogels and hydrogels it was observed that the elastic modulus (G′) was greater than loss modulus (G″), demonstrating the dominance of elastic nature over viscous nature. The stress sweep plots of the gels (Figure 3A,B) demonstrate a slightly more mechanically robust network (tolerance strain 41%) for the C200 organogel compared to its DC200 hydrogel counterpart (tolerance strain 34%). The frequency sweep measurements of the organogels and hydrogels were conducted at a fixed strain 2.0% which is sufficiently low strain for the deformation of the gels to happen. The results presented in Figure 3C,D display frequency independent behavior of G´ over the experimental frequency range suggesting polymeric network formation with covalent cross-linking. To determine the variation of mechanical properties at different Ac-glu-HEMA/cross-linker ratios, the frequency sweep measurements were performed for the gels having different cross-linker ratios. It was observed that in both the cases of organogels and hydrogels, the magnitude of G´ decreases gradually with increasing Ac-glu-HEMA/cross-linker ratio. In other words, more robust gels could be observed with the gels possessing high cross-link density. In addition to this, the strengthening of the 3D cross-linked network for the organogels over hydrogels can be further supported by results from the frequency sweep experiments. The magnitude of the G´ values for the organogels were somewhat higher than the corresponding hydrogels which is likely to be because the water very effectively solvates the polymer backbones of the hydrogels.

### 2.3. Gel Swelling in Various Solvents

**Figure 4:**
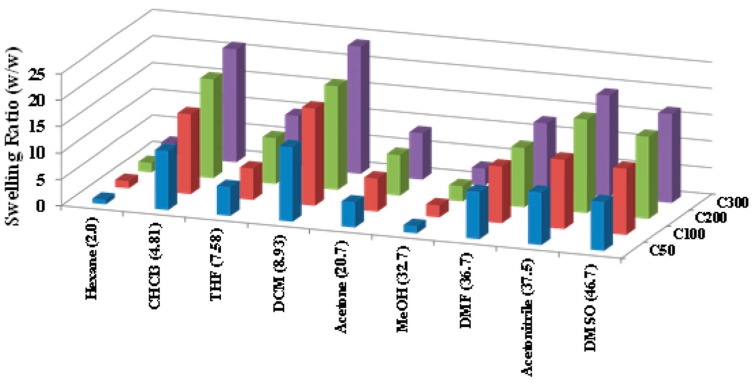
Swelling behavior of different carbohydrate based organogels in various organic solvents.

Various factors play a role in deciding the swelling ability of a polymer gel, including: the structure of the polymer chains, the cross-linking density of the 3D network of polymer chains, and solvent–solute interactions [28,29,30,31,32,33,34,35,36,37]. To determine the amount of solvent uptake by the organogels, their swelling behaviors were measured in different organic solvents of various dielectric constants (ɛ), hexanes (ɛ = 1.88), CHCl_3_ (ɛ = 4.81), THF (ɛ = 7.5), DCM (ɛ = 9.1), acetone (ɛ = 20.7), methanol (ɛ = 33), DMF (ɛ = 36.7), acetonitrile (ɛ = 37.5), DMSO (ɛ = 46.7) and water (ɛ = 80.0). The results are summarized in Figure 4 which demonstrates that the swelling was greatest in DCM (i.e., not the solvent with the highest ɛ). The swelling ratios of the organogels were also measured at varying monomer/cross-linker ratios (Figure 4 and Table 2) demonstrate that the swelling ability of the organogels increased from C50 to C300, because increasing monomer/cross-linker ratio leads to a corresponding decrease in cross-linking density in the 3D matrix enabling uptake of a higher volume of solvent. It is noteworthy that in strongly polar solvents like methanol the swelling efficiency of the organogels is very low and negligible in water, which demonstrates the dielectric constant of a solvent is not suitable to describe the swelling in these systems [28,29,30,31,32,33,34,35,36,37].

Solvent–solute interactions have a large effect upon the supramolecular chemistry of a system and there is considerable interest quantitatively understanding the role of solvents on the gelation phenomenon [28,29,30,31,32,33,34,35,36,37]. A solvent’s properties on the macroscopic level (i.e., refractive index, density, etc.) or molecular level (i.e., intermolecular forces, solvation, etc.) can be quantified. Bulk properties include the dielectric constant (ε) and Reichardt’s parameter (E_T_); see Table A1 [28,29,30,31]. Molecular level solvent properties include the Kamlet–Taft parameters (Table A1), π* (a generalized polarity parameter), α (ability to donate hydrogen bonds), and β (ability to accept hydrogen bonds), and Hildebrand solubility parameters (Table A2) [28,29,30,31], δ (expressed in terms of Hildebrand’s total cohesion parameter (δ_t_), the total solubility parameter (δ_o_), which is described by the dispersion, polar, and hydrogen bonding parameters, δ_d_, δ_p_, and δ_h_, respectively; and the parameters δ_p_ and δ_h_ are described in terms of a “combined polar solubility parameter”, δ_a_). Solvent effects governing the hierarchical assembly of polymer gels in a variety of different solvents have been studied, and the precise hydrogen-bonding nature of the solvent (hydrogen bond donors and acceptors) can be insightful to fully understand the solvent–solute interactions that govern the swelling of gels [28,29,30,31,32,33,34,35,36,37]. Akin to the dielectric constant (ɛ), there was no correlation of the normalized Reichardt E_T_ value to the swell ratio, nor was there a correlation of the swell ratio to the Kamlet–Taft parameters π*, α or β; nor was there a clear correlation of the swell ratio to Hildebrand’s solubility parameters. This demonstrates the solvent parametric approach is not universally applicable to describe the swelling of all gels in various solvents [28,29,30,31].

**Figure 5:**
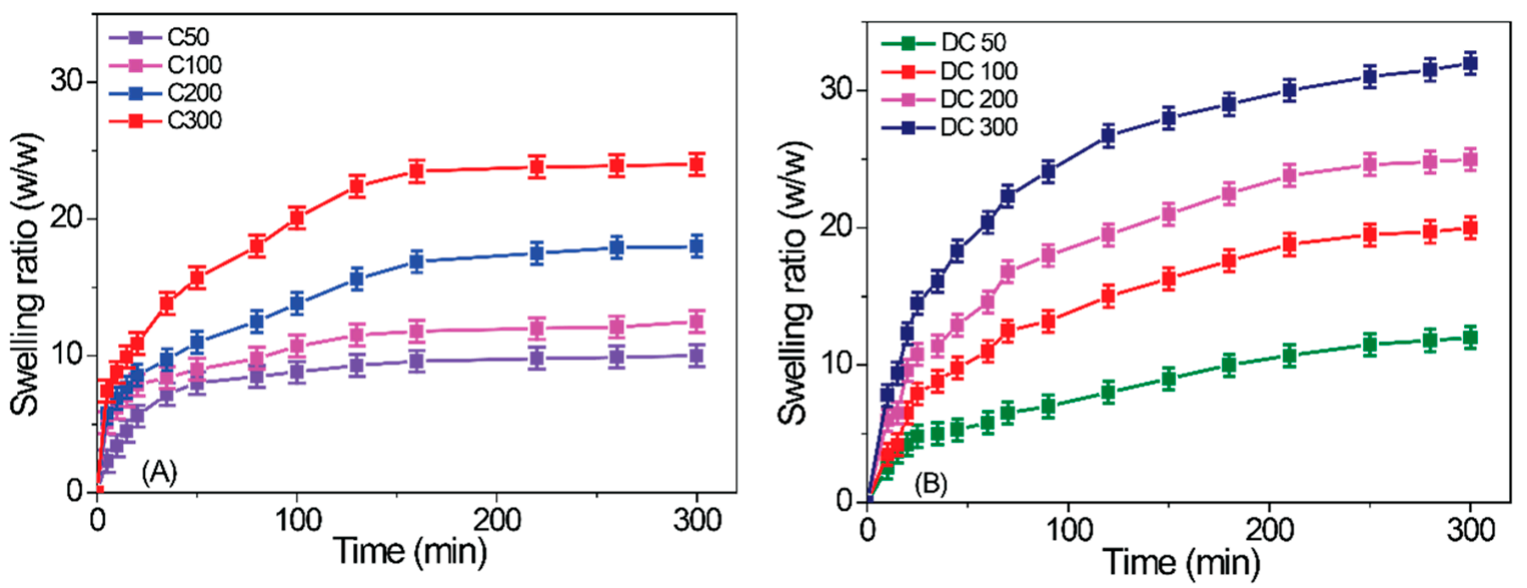
Variation of swelling ratio with time for different carbohydrate-based gels: (**A**) organogels in dichloromethane (DCM) and (**B**) hydrogels in aqueous medium (pH 6.8).

**Figure A2:**
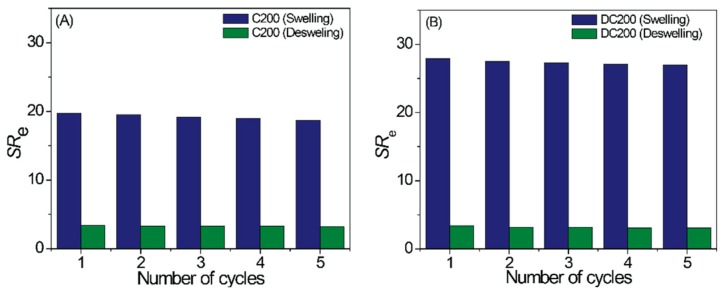
Swelling–deswelling cycles. (**A**) C200 in DCM. (**B**) DC200 in deionized water.

The swelling kinetics of both organogels and hydrogels were investigated. The measurements on organogels were performed in DCM, whereas those for hydrogels in water, respectively. Figure 5A shows that C50, C100, C200 and C300 organogel reached its maximum swelling at 130, 158, 161, 170 min, respectively. Similarly, Figure 5B displays that DC50, DC100, DC200 and DC300 hydrogels attained its maximum swelling degree in 210, 226, 240, 269 min, respectively. The swelling/deswelling capability of depends on the crosslinking ratio, the less cross-linked gel takes less time to swell than the more highly cross-linked gels. Reversible swelling and de-swelling of gels is a useful property for a variety of applications (e.g., coatings on medical devices) [38,39,40]. Consequently the ability of the organogels to reversibly swell/dry (using DCM as the organic solvent) were studied; similarly, we studied the ability of the hydrogels to reversibly swell/dry (using water), in both cases observing this to be reversible for five rounds of swelling/drying (Figure A2).

### 2.4. Uptake and Release of Species from Hydrogels

The uptake of various species was studied to assess the potential of the hydrogels for environmental applications (e.g., waste water purification [41,42,43,44,45,46,47]) or biomedical applications (e.g., wound dressings [3,48,49,50,51,52]).

**Figure 6:**
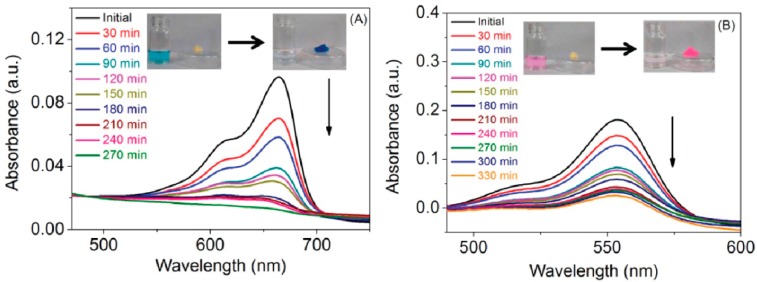
UV–Vis spectra of methylene blue (MB) (**A**) and rhodamine-B (RhB) (**B**) adsorption by DC200 gels at different time intervals.

The uptake of two water soluble dyes (methylene blue (MB), rhodamine B (RhB)) was studied quantitatively by UV–Vis measurements at various time points (Figure 6). There was significant uptake of the dyes within a few hours (90%–92% of MB and 81%–83% of RhB), and we therefore believe that such gels have potential for application in waste water purification (e.g., removing dyestuffs from the waste streams of industrial plants), or inclusion in wound dressings where uptake of wound exudate is important.

**Figure 7:**
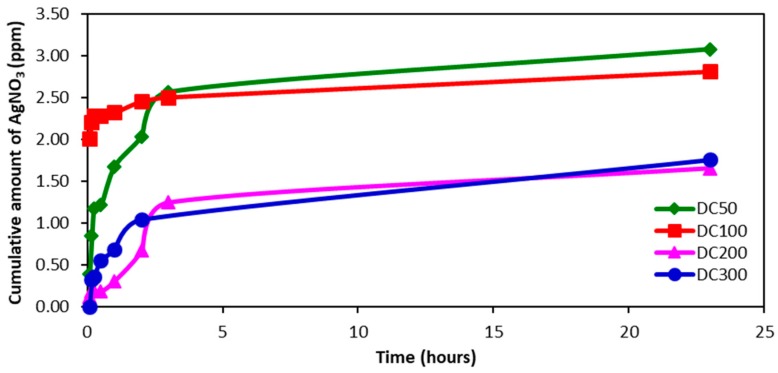
Cumulative release of Ag^+^ (ppm) from the hydrogels.

The potential of the gels to release bioactive species (e.g., antimicrobial species including metal ions) may be useful for both environmental and biomedical applications. The gels swell in water and aqueous solutions of AgNO_3_ (Table 3), highlighting their potential to absorb and act as reservoirs for the subsequent release of Ag^+^ which has antimicrobial activity (Table 3 and Figure 7); the maximum amount of Ag^+^ was released from DC50, followed by DC100, DC300 and DC200.

**Figure 8:**
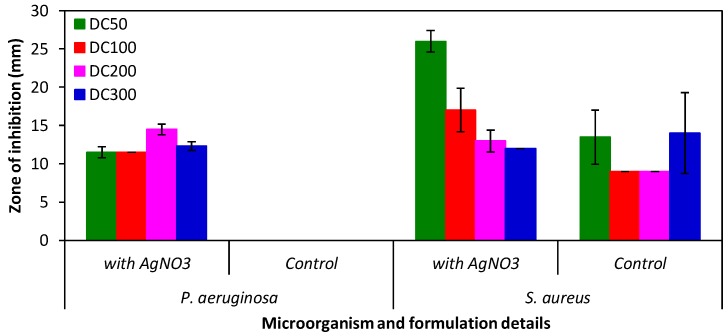
Antimicrobial potential of AgNO_3_ loaded hydrogels indicated as the zone of inhibition (ZOI) against *P. aeruginosa* (Gram negative) and *S. aureus* (Gram positive). Diameter of the well = 5 mm; *n* = 3; error bars = standard deviation.

Consequently, the potential of the hydrogels to deliver Ag^+^ was assessed by studying their antimicrobial activity against two representative wound infecting microorganisms, *S. aureus* (Gram positive) and *P. aeruginosa* (Gram negative) (Figure 8). DC50 and DC100 were more active against *S. aureus* (ZOI: DC50 = 26.0 mm and DC100 = 17.0 mm) than *P. aeruginosa* (ZOI: 11.5 mm for both DC50 and DC100). In contrast, DC200 showed a slight improvement in activity against *P. aeruginosa* (ZOI = 14.5 mm) when compared to *S. aureus* (ZOI = 13.0 mm), whilst the antimicrobial activity was relatively similar for DC300 (ZOI: *P. aeruginosa* ZOI = 12.3 mm and *S. aureus* ZOI = 12.0 mm). In control studies, hydrogels hydrated with SDW showed no activity against *P. aeruginosa* but exhibited some antimicrobial activity against *S. aureus*. Low et al. have previously reported that the minimum bactericidal concentration (MBC) of AgNO_3_ against *P. aeruginosa* was 1.6 × 10^−3^% *w*/*v* (equivalent to 10.16 ppm of active Ag^+^) and 5.1 × 10^−3^% *w*/*v* against *S. aureus* (equivalent to 32.39 ppm of active Ag^+^) [53]. While the release of Ag^+^ from 25 mg of gel was much lower when compared to the reported MBC of AgNO_3_, it would be possible to use larger quantities of the gel to enable the delivery of a therapeutically relevant microbicidal concentration of Ag^+^ to improve efficacy.

The variation in the observed antimicrobial activity is attributed to the difference in complexity and composition of the cellular membrane/wall between the Gram positive and negative bacteria [54,55]. We attribute the somewhat lower antimicrobial activity of the gels against *P. aeruginosa* to the known resistive characteristics of Gram negative bacteria because of their impermeable outer membrane barrier, along with other potential defensive features such as efflux pumps, regulation of enzymes and outer membrane porins to degrade or inhibit the activity of the antimicrobial agents [56,57]. The current results demonstrated the feasibility of using these gels for biomedical applications. Further studies to investigate the potential of incorporating a range of different antimicrobial agents (e.g., essential oils (TTO) and/or metal ions (Ag^+^)) within the gels, and imparting novel chemistry in the polymers to generate smart responsive hydrogels has significant potential for the management of wounds.

## 3. Conclusions

In summary, we have demonstrated synthesis of *β*-D-glucose pentaacetate containing polymeric organogels via RAFT technique. Transformation of organogels to corresponding hydrogels was achieved by simple deprotection of the acetyl groups in the organogel matrix yielding hydroxyl counterpart in the hydrogel. The swelling ability of the organogels was checked in different solvents with varying dielectric constant and highest swelling was observed in DCM, which also varied with the monomer/cross-linker ratio in the gel network. FE-SEM experiment confirmed porous network formation in the gel matrix and the size of the pores increased with increasing monomer/cross-linker ratio. Gel stiffness as observed from rheological measurements decreased with increasing monomer/cross-linker ratio. Thus, gel stiffness, network morphology, and solvent uptake capacity could be controlled by varying monomer/cross-linker ratio. The versatility and potential of using these gels, both for environmental and biomedical applications, is highlighted herein, and further developments will include stimuli-responsive moieties to generate instructive biomaterials.

## 4. Materials and Methods 

### 4.1. Materials

Sodium methoxide (NaOMe), *β*-D-glucose pentaacetate (98%), 4-dimethylaminopyridine (DMAP, 99%), anhydrous *N,N′*-dimethylformamide (DMF, 99.9%), *N,N′*-dicyclohexylcarbodiimide (DCC, 99%), boron trifluoride diethyl etherate (BF_3_·Et_2_O, 46.5%), methylene blue (MB), rhodamine-B (RhB) and 2-hydroxyethyl methacrylate (HEMA, 97%) were purchased from Sigma-Aldrich, India, and used without any further purification. The 2,2’-azobisisobutyronitrile (AIBN, Sigma-Aldrich, India, 98%) was purified by recrystallization twice from methanol. The di(ethylene glycol) dimethacrylate (DEGDMA, 95%, Sigma Aldrich, Mumbai, Maharashtra, India) was purified by passing through a basic alumina column to remove any residues of potential inhibitors. The 4-cyano-4-(dodecylsulfanylthiocarbonyl)sulfanylpentanoic acid (CDP) [24] and *β*-D-glucose pentaacetate containing methacrylate monomer (Ac-glu-HEMA) [58]. Deuterated CHCl_3_ (CDCl_3_, 99.8% D) was purchased from Cambridge Isotope Laboratories Inc., Andover, MA, USA. Solvents such as hexanes (mixture of isomers), acetone, ethyl acetate (EtOAc), tetrahydrofuran (THF), methanol (CH_3_OH), chloroform (CHCl_3_), dichloromethane (DCM), acetonitrile (CH_3_CN) and dimethyl sufoxide (DMSO) were purchased from Sigma-Aldrich.

Nutrient broth and agar (tryptone soya broth (TSB) and tryptone soya agar (TSA); Lab M, Lancaster, UK) were prepared according to the manufacturer’s recommendations and sterilized by autoclaving at 121 °C for 15 min. Similarly, ¼ Ringers solution (Lab M, Lancaster, UK) was prepared according to manufacturer’s recommendation and sterilized by autoclaving. Silver nitrate solution (0.0508% *w*/*v* AgNO_3_; 99.85% purity; Acros Organics, Geel, Belgium) solutions were aseptically prepared in sterile distilled water for each experiment. Tea tree oil (TTO) was purchased from FreshSkin Beauty Limited, Nottingham, UK and polyvinyl alcohol (PVA, 87–90% hydrolysed, average molecular weight 30–70 kDa) was purchased from Sigma Aldrich, Gillingham, UK. The TTO:PVA emulsion was prepared by sonicating (Bandelin Sonopuls HD2200; Bandelin Electronic, Berlin, Germany) TTO and 10% *w*/*v* PVA at a ratio of 40:60% *v*/*v* for 1 min.

### 4.2. Analytical Chemistry

UV–Vis spectra were recorded using a Perkin-Elmer Lambda 35 UV–Vis spectrophotometer. FTIR spectra of KBr pellets of substances were recorded using a Perkin–Elmer spectrum 100 FTIR spectrometer. ^1^H NMR spectra were recorded on a Bruker Avance III 500 spectrometer at 25 °C. Solid-state ^13^C cross-polarization/magic-angle spinning (CP/MAS) NMR was also carried out in the same Bruker Avance III 500 spectrometer; the broad band channel was tuned to 125 MHz (the resonance frequency of ^13^C), and a 4 mm MAS probe was used for the experiment at a spinning speed of 10 KHz; a typical ^13^C value of pulse length was used for 4 µs and relaxation delay of 20 s was used. 

### 4.3. Preparation of Organogels

Carbohydrate containing chemically cross-linked gels were synthesized in the presence of AIBN as a radical source, CDP as a chain transfer agent (CTA), and DEGDMA as a cross-linker in DMF at 70 °C. Typically, Ac-glu-HEMA (1.00 g, 2.17 mmol) [26], DEGDMA (10.5 mg, 0.434 µmol), CDP (4.38 mg, 0.108 µmol), AIBN (0.53 mg, 3.25 µmol, from stock solution) and 0.4 mL DMF were taken in a 20 mL septa sealed vial equipped with a magnetic stir bar. Then, the reaction vial was purged with dry nitrogen for 20 min to make the system inert and placed in a preheated reaction block at 70 °C for 24 h. Stirring was stopped when a viscous gel was formed to eliminate bubble entrapment inside the gels. After 24 h, the reaction was quenched by putting the vial in an ice water bath and exposing the mixture to air. Semi-solid crude gels were collected after breaking the polymerization vial very carefully. Then, it was placed in a 250 mL beaker, washed/dialyzed against acetone (3 × 200 mL) and hexanes (3 × 200 mL) to remove unreacted monomers, DMF and other impurities. Finally, all gels were dried in a fume hood for 6 h, followed by drying under high vacuum at 40 °C for two days.

### 4.4. Preparation of Hydrogels

The acetyl groups on the organogels were removed by using NaOMe to obtain hydrogels with hydroxyl (-OH) functionality. In a typical example, 100 mg of protected gel was swelled in DCM then immersed in 3 mL NaOMe for 24 h. Then the swelled gel was washed 5–6 times with methanol, dried in a fume hood for 6 h, followed by drying under high vacuum at 45 °C for two days.

### 4.5. Gel Swelling Studies

The equilibrium swelling ratio (SRe) of the gels was investigated gravimetrically at room temperature. A small amount of dry organogel was immersed in different solvents (e.g., hexanes, CHCl_3_, DCM, DMSO, THF, CH_3_OH, de-ionized (DI) water, sterile distilled water (SDW), silver nitrate solution (0.0508% *w*/*v*), etc.) for 24 h, after which they were removed from the container, the surface of the gel wicked with tissue paper and weighed. The SRe value was calculated using Equation (1):

(1) $$Equation\;swelling\;ratio\;\left(SR_{E}\right)=\;\frac{\left(W_{s}-W_{d}\right)}{W_{d}}$$

where *W_d_* and *W_s_* are the mass of the dried and swollen gels, respectively. The retention kinetics of various gels were also measured by gravimetric analysis at room temperature. To measure the solvent retention capability, a small piece of the fully swollen gel was kept in open air and weighed the mass at different time interval. Solvent retention was determined from Equation (2):

(2) $$Solvent\;retention=\frac{\left(W_{s}-W_{d}\right)}{\left(W_{0}-W_{d}\right)}$$

where *W_s_* is the weight of gel at time *t*, *W*_0_ is the weight of swollen gel at time *t* = 0 and *W_d_* is the weight of the dry gel. For re-swelling tests gels were repetitively swollen and dried.

### 4.6. Rheology

To investigate the mechanical strength of the gels, we performed rheological measurements using a TA-ARG2 rheometer equipped with 40 mm diameter steel parallel plate with plate gap of 1.0 mm at 25 °C. Storage modulus (G′) and loss modulus (G″) were recorded in the linear viscoelastic regime at a shear strain of γ = 1%, with angular frequency sweep in the range of 0.1 to 100 rad/s. All the gel samples were prepared according to our previously reported procedure [59].

### 4.7. Field Emission Scanning Electron Microscopy (FE-SEM) Analysis

A small piece of dried gel sample was immersed in DCM (for organogels) or deionised water (for hydrogels) for overnight. The swelled organogels were cross sectioned with a surgical knife and dried over silicon-wafer for 12 h under high vacuum. The hydrogels were frozen using liquid nitrogen and freeze dried using a lyophilizer (Operon, Gimpo, Gyeonggi, Korea) at −50 °C. Finally, the dry hydrogel sample was sputter coated with a very thin layer of gold-palladium (Au-Pd) alloy for 1 min and examined by FE-SEM (Carl Ziess Supra SEM instrument, Oberkochen, Germany).

### 4.8. Dye Uptake Study

Dry DC200 gel (25 mg) was added to 10 mL of acidic (pH 6) aqueous solutions of either MB (200 mg/L) or RhB (300 mg/L) and slowly stirred, after which UV–Vis spectra of the solution (MB: λ_max_ = 664 nm. RhB λ_max_ = 554 nm) were measured at various time points. Rearrangement of the Lambert–Beer law (Equation (3)) enabled calculation of the concentration of the dyes in solution:

*A* = *ε.c.l* (3)

where *A* is the absorbance, *ε* is the molar extinction coefficient, *c* is the concentration and *l* is the path length. The % adsorption was subsequently calculated using Equation (4):

(4) $$Percentage\;adsorption\;\left(\%\right)=\frac{\left(C_{0}-C_{t}\right)}{C_{0}}\times 100\%$$

where, *C*_0_ is the initial concentration (mg/L) and *C_t_* is the concentration of dye in solution at various time points (an average of three readings). Similarly, dye removal studies were performed using Equation (4).

### 4.9. Inductively Coupled Plasma (ICP) Analysis of Ag^+^ Release

The gels hydrated in AgNO_3_ were incubated in 30 mL sterile distilled water (SDW) and samples 3 mL samples were drawn and replaced with 3 mL of fresh SDW at 5 min intervals for the first 15 min followed by sampling at 0.5, 1, 2, 3 and 23 h. The collected samples were diluted with 6 mL of SDW and subjected to inductively coupled plasma (ICP) analysis to determine the silver ion (Ag^+^) release using a Spectro Ciros Charged Coupled Device (CCD; Spectro, Kleve, Germany).

### 4.10. Antimicrobial Studies—Zone of Inhibition (ZOI)

Overnight cultures of *P. aeruginosa* (NCIB 8295) or *S. aureus* (NCIB 6571) were prepared by aseptically inoculating 50 mL of sterile TSB and incubating overnight in an orbital shaker at 37 °C (Series 25; New Brunswick Scientific Co. Inc., Edison, NJ, USA). The density of the overnight cultures was determined using a standard Miles and Misra technique [60].

The potential antimicrobial effect of gels hydrated with AgNO_3_ solutions were tested using an adapted standard well diffusion method. The starter inoculating cultures used for the antimicrobial zone of inhibition (ZOI) studies were approximately 1 × 10^7^ colony forming units (CFU)/mL. The overnight cultures were aseptically swabbed on the surface of sterile TSA according to the recommendations in the BSAC disc diffusion method [61]. Briefly, sterile cotton swabs were dipped into the overnight cultures and the excess liquid were removed by pressing against the side of the container. The inoculums were aseptically swabbed evenly over the entire surface of the TSA in three different directions. Following that, a 5 mm diameter well was aseptically bored in the middle of the agar plate using a metal borer. AgNO_3_ hydrated gels (0.025 g of formulations C50, C100, C200 and 0.2 g of formulation C300) were placed in the well. For formulations C50, C100 and C200, 40 μL of sterile ¼ Ringers solution were aseptically added into the well because the hydrogels did not fill up the well. This was done in triplicate. Similarly, controls were set up by replacing the hydrogels with SDW hydrated gels. The ZOI were measured after incubating the plates overnight in a static incubator at 37 °C.

## Figures and Tables

**Table 1:** Conditions used for RAFT synthesis of organogels.

Gel	Ac-glu-HEMA/DEGDMA/CDP/AIBN	Gelation Time (min)	Monomer Conversion after 24 h (%)
C50	50/2/0.5/0.15	90	77
C100	100/2/0.5/0.15	105	75
C200	200/2/0.5/0.15	135	80
C300	300/2/0.5/0.15	148	79

**Table 2:** Swelling ratios (*w*/*w*) of carbohydrate based organogels after 24 h in different organic solvents (the tea tree oil (TTO):polyvinyl alcohol (PVA) is an emulsion of 40% TTO, 60% water (10% *w*/*v* PVA)).

Gel	Isopropyl Palmitate	Isopropyl Myristate	Olive Oil	SSC	SSH	TTO:PVA
C50	0.12	0.08	0.15	0.21	0.25	1.59
C100	0.30	0.18	0.76	0.42	0.56	1.09
C200	0.39	1.10	1.67	1.40	0.87	1.04

**Table 3:** Swell ratio (*w*/*w*) of hydrogels after 24 h in sterile distilled water (SDW) or 0.0508% AgNO_3_ solution.

Hydrogel	SDW	AgNO_3_ Solution	Ag^+^ Release (ppm)
DC50	1.60	3.10	3.08
DC100	2.17	1.44	2.81
DC200	2.35	2.69	1.66
DC300	5.28	3.62	1.76

## Appendix A

**Table A1:** Dielectric constant (ε), normalized Reichardt E_T_ values and Kamlet–Taft parameters for a selection of solvents investigated in this paper.

Gel	ε	E_T_^N^	α	β	π*
Hexane	2.00	0.009	0.00	0.00	−0.08
Chloroform	4.80	0.259	0.44	0.00	0.69
THF	7.58	0.207	0.00	0.55	0.55
Dichloromethane	8.93	0.309	0.30	0.00	0.73
Acetone	20.70	0.355	0.08	0.48	0.71
Methanol	32.70	0.762	0.93	0.62	0.60
DMF	36.70	0.386	0.00	0.76	0.88
Acetonitrile	37.50	0.460	0.19	0.31	0.75
DMSO	46.70	0.444	0.00	0.76	1.00
Water	78.3	1.100	1.15	0.15	1.10

Dielectric constant (ε). Reichardt’s parameter (E_T_). Kamlet–Taft parameters: α (ability to donate hydrogen bonds), β (ability to accept hydrogen bonds), and π* (a generalized polarity parameter).

**Table A2:** Hildebrand solvent parameters for a selection of solvents investigated in this paper (-, not available).

Gel	δ_t_	δ_o_	δ_d_	δ_p_	δ_h_	δ_a_
Hexane	14.9	-	14.9	-	-	2.1
Chloroform	18.9	9.30	8.70	1.50	2.80	3.18
THF	18.6	9.50	8.20	2.80	3.90	4.80
Dichloromethane	20.2	9.90	8.90	3.10	3.00	4.31
Acetone	19.6	10.4	13.9	-	-	-
Methanol	29.7	10.0	12.7	6.00	10.90	17.0
DMF	24.1	12.7	16.2	-	-	-
Acetonitrile	24.7	16.8	13.3	-	-	-
DMSO	24.5	12.5	17.2	-	-	-
Water	47.9	-	12.9	-	-	-

Hildebrand solubility parameters, δ (expressed in terms of Hildebrand’s total cohesion parameter (δ_t_), the total solubility parameter (δ_o_), which is described by the dispersion, polar, and hydrogen bonding parameters, δ_d_, δ_p_, and δ_h_, respectively; and the parameters δ_p_ and δ_h_ are described in terms of a “combined polar solubility parameter”, δ_a_).
